# Supplementary material for: Effects of Antiviral Therapy and Glucocorticoid Therapy on Fever Duration in Pediatric Patients with Influenza
Source: Medicina (Kaunas). 2021 Dec 20;57(12):1385. doi: 10.3390/medicina57121385 (PMC8707365; doi:10.3390/medicina57121385)
Supplement: Supplementary file 1 [file medicina-57-01385-s001.zip › medicina-1470014-supplementary.pdf]

Table S1. Results of the multivariate Cox regression analysis according to clinical diagnosis.

Upper respiratory tract infection on admission			
Factor	Hazard ratio	95% Confidence interval	*p*-value
Female (vs. male)	1.057	0.887–1.261	0.538
Age, years	0.955	0.934–0.977	<0.001
Pre-admission fever, days	0.880	0.828–0.936	<0.001
Influenza B (vs. influenza A)	1.427	1.178–1.727	<0.001
Type of administered treatment			
No therapy	reference		
NAI therapy	1.007	0.726–1.395	0.967
GC therapy	0.993	0.698–1.414	0.971
Both therapies	1.010	0.739–1.381	0.949
Lower respiratory tract infection on admission			
Factor	Hazard ratio	95% Confidence interval	*p*-value
Female (vs. male)	1.098	0.678–1.779	0.705
Age, years	0.947	0.878–1.020	0.153
Pre-admission fever, days	0.880	0.787–0.984	0.025
Influenza B (vs. influenza A)	0.663	0.411–1.070	0.092
Type of administered treatment			
No therapy	reference		
NAI therapy	0.591	0.104–3.356	0.553
GC therapy	0.388	0.086–1.748	0.217
Both therapies	0.419	0.094–1.020	0.257

NAI, neuraminidase inhibitor; GC, glucocorticoid.

Table S2. Results of the multivariate Cox regression analysis according to influenza virus subtype.

Influenza A virus infection			
Factor	Hazard ratio	95% Confidence interval	*p*-value
Female (vs. male)	1.242	1.013–1.524	0.037
Age, years	0.973	0.948–1.000	0.046
Pre-admission fever, days	0.839	0.779–0.803	<0.001
LRI on admission (vs. URI)	0.895	0.649–1.235	0.501
Type of administered treatment			
No therapy	reference		
NAI therapy	0.799	0.531–1.203	0.282
GC therapy	0.705	0.462–1.076	0.106
Both therapies	0.802	0.544–1.181	0.264
Influenza B virus infection			
Factor	Hazard ratio	95% Confidence interval	*p*-value
Female (vs. male)	1.242	0.620–1.092	0.176
Age, years	0.929	0.896–0.964	<0.001
Pre-admission fever, days	0.896	0.829–0.969	0.006
LRI on admission (vs. URI)	0.381	0.254–0.572	<0.001
Type of administered treatment			
No therapy	reference		
NAI therapy	1.114	0.658–1.883	0.689
GC therapy	1.031	0.583–1.821	0.918
Both therapies	0.982	0.591–1.631	0.945

LRI, lower respiratory tract infection; URI, upper respiratory tract infection; NAI, neuraminidase inhibitor; GC, glucocorticoid.

Table S3. Results of the multivariate Cox regression analysis according to the pre-admission fever duration.

Pre-admission fever ≤2 days			
Factor	Hazard ratio	95% Confidence interval	*p*-value
Female (vs. male)	1.063	0.886–1.276	0.515
Age, years	0.962	0.939–0.985	0.001
Influenza B (vs. influenza A)	1.279	1.052–1.555	0.014
LRI on admission (vs. URI)	0.673	0.498–0.911	0.010
Type of administered treatment			
No therapy	reference		
NAI therapy	1.005	0.693–1.458	0.980
GC therapy	1.042	0.705–1.538	0.839
Both therapies	1.038	0.727–1.481	0.836
Pre-admission fever >2 days			
Factor	Hazard ratio	95% Confidence interval	*p*-value
Female (vs. male)	0.816	0.536–1.242	0.342
Age, years	0.930	0.883–0.979	0.006
Influenza B (vs. influenza A)	1.157	0.776–1.727	0.472
LRI on admission (vs. URI)	0.556	0.347–0.891	0.015
Type of administered treatment			
No therapy	reference		
NAI therapy	1.134	0.585–2.198	0.710
GC therapy	0.688	0.339–1.397	0.301
Both therapies	0.852	0.457–1.590	0.614

LRI, lower respiratory tract infection; URI, upper respiratory tract infection; NAI, neuraminidase inhibitor; GC, glucocorticoid.

Table S4. Results of the multivariate Cox regression analysis according to patient age.

Age of <5 years			
Factor	Hazard ratio	95% Confidence interval	*p*-value
Female (vs. male)	0.978	0.783–1.224	0.850
Pre-admission fever, days	0.907	0.848–0.970	0.004
Influenza B (vs. influenza A)	1.381	1.082–1.764	0.010
LRI on admission (vs. URI)	0.651	0.486–0.873	0.004
Type of administered treatment			
No therapy	reference		
NAI therapy	1.070	0.687–1.664	0.768
GC therapy	1.002	0.639–1.570	0.993
Both therapies	0.953	0.630–1.443	0.822
Age of 5–9 years			
Factor	Hazard ratio	95% Confidence interval	*p*-value
Female (vs. male)	1.114	0.825–1.504	0.482
Pre-admission fever, days	0.801	0.710–0.904	<0.001
Influenza B (vs. influenza A)	1.321	0.956–1.825	0.091
LRI on admission (vs. URI)	0.513	0.287–0.915	0.024
Type of administered treatment			
No therapy	reference		
NAI therapy	0.810	0.449–1.460	0.483
GC therapy	0.833	0.453–1.531	0.557
Both therapies	1.035	0.599–1.789	0.900
Age of ≥10 years			
Factor	Hazard ratio	95% Confidence interval	*p*-value
Female (vs. male)	1.479	0.915–2.392	0.110
Pre-admission fever, days	0.742	0.636–0.866	<0.001
Influenza B (vs. influenza A)	0.880	0.532–1.456	0.619
LRI on admission (vs. URI)	0.737	0.309–1.757	0.491
Type of administered treatment			
No therapy	reference		
NAI therapy	0.587	0.237–1.456	0.250
GC therapy	0.388	0.129–1.168	0.092
Both therapies	0.551	0.230–1.319	0.181

LRI, lower respiratory tract infection; URI, upper respiratory tract infection; NAI, neuraminidase inhibitor; GC, glucocorticoid.
